# Prognostic Significance of Nonischemic Myocardial Fibrosis in Patients With Normal LV Volumes and Ejection-Fraction

**DOI:** 10.1016/j.jcmg.2021.05.016

**Published:** 2021-12

**Authors:** Amrit S. Lota, Adam Tsao, Ruth Owen, Brian P. Halliday, Dominique Auger, Vassilios S. Vassiliou, Upasana Tayal, Batool Almogheer, Silvia Vilches, Amer Al-Balah, Akhil Patel, Florence Mouy, Rachel Buchan, Simon Newsome, John Gregson, James S. Ware, Stuart A. Cook, John G.F. Cleland, Dudley J. Pennell, Sanjay K. Prasad

**Affiliations:** a Cardiovascular Research Centre & Cardiovascular Magnetic Resonance Unit, Royal Brompton and Harefield Hospitals NHS Foundation Trust, London, United Kingdom; b National Heart & Lung Institute, Imperial College London, London, United Kingdom; c Imperial College London Medical School, London, United Kingdom; d London School of Hygiene and Tropical Medicine, London, United Kingdom; e Norwich Medical School, University of East Anglia, Norwich, United Kingdom; f Brighton and Sussex Medical School, Brighton, United Kingdom; g MRC London Institute of Medical Sciences, London, United Kingdom; h National Heart Centre Singapore, Singapore; i Robertson Centre for Biostatistics, University of Glasgow, Glasgow, United Kingdom

**Keywords:** cardiovascular magnetic resonance, late gadolinium enhancement, myocardial fibrosis, myocarditis, sudden cardiac death, BSA, body surface area, CV, cardiovascular, ICD, implantable cardioverter-defibrillator, LGE, late gadolinium enhancement, LV, left ventricular, LVEF, left ventricular ejection fraction, MRI, cardiovascular magnetic resonance, SCD, sudden cardiac death

## Abstract

**Objectives:**

This study aims to investigate the prognostic significance of late gadolinium enhancement (LGE) in patients without coronary artery disease and with normal range left ventricular (LV) volumes and ejection fraction.

**Background:**

Nonischemic patterns of LGE with normal LV volumes and ejection fraction are increasingly detected on cardiovascular magnetic resonance, but their prognostic significance, and consequently management, is uncertain.

**Methods:**

Patients with midwall/subepicardial LGE and normal LV volumes, wall thickness, and ejection fraction on cardiovascular magnetic resonance were enrolled and compared to a control group without LGE. The primary outcome was actual or aborted sudden cardiac death (SCD).

**Results:**

Of 748 patients enrolled, 401 had LGE and 347 did not. The median age was 50 years (interquartile range: 38-61 years), LV ejection fraction 66% (interquartile range: 62%-70%), and 287 (38%) were women. Scan indications included chest pain (40%), palpitation (33%) and breathlessness (13%). No patient experienced SCD and only 1 LGE+ patient (0.13%) had an aborted SCD in the 11th follow-up year. Over a median of 4.3 years, 30 patients (4.0%) died. All-cause mortality was similar for LGE+/- patients (3.7% vs 4.3%; *P =* 0.71) and was associated with age (HR: 2.04 per 10 years; 95% CI: 1.46-2.79; *P <* 0.001). Twenty-one LGE+ and 4 LGE- patients had an unplanned cardiovascular hospital admission (HR: 7.22; 95% CI: 4.26-21.17; *P <* 0.0001).

**Conclusions:**

There was a low SCD risk during long-term follow-up in patients with LGE but otherwise normal LV volumes and ejection fraction. Mortality was driven by age and not LGE presence, location, or extent, although the latter was associated with greater cardiovascular hospitalization for suspected myocarditis and symptomatic ventricular tachycardia.

There has been rapid growth in the adoption of cardiovascular magnetic resonance (MRI) imaging for diagnostic evaluation, surveillance, and assessment of treatment response across the spectrum of cardiovascular (CV) disease. Appropriate-use criteria highlight the evolution in complexity and capability of MRI to support clinical decision-making (1,2). Replacement fibrosis (scar) identified by late gadolinium enhancement (LGE) indicates an adverse prognosis in many conditions, which are all characterized by abnormal left ventricular (LV) volumes and/or LV ejection fraction (LVEF) (3, 4, 5, 6). However, myocardial fibrosis remains a powerful predictor of sudden cardiac death (SCD) even when the severity of LV dysfunction is only modest (7).

Increasing numbers of individuals are identified with normal LV volumes, wall thickness, and LVEF and previously unrecognized myocardial fibrosis. In one of many series, subendocardial fibrosis suggesting myocardial infarction was present in 17% of people older than 67 years of age with incremental prognostic value beyond standard clinical predictors including LVEF (8, 9, 10, 11). However, there is a paucity of data on the prognostic significance of nonischemic fibrosis in the midwall and/or subepicardium of people with normal LV volumes, wall thickness, and LVEF. Fibrosis represents the final common pathway of injury from a diverse range of diseases and insults (12). Whether nonischemic fibrosis is a risk factor for SCD in the absence of other structural markers of disease such as LV dysfunction/dilation is unknown. Moreover, the etiology of midwall/subepicardial fibrosis is often unclear and ascribed to remote events, such as previous myocarditis. Uncertainty surrounding the clinical significance and management of such cases leads to conflicting advice, such as refraining from high-intensity exercise, for which there is no evidence of benefit, multiple investigations at considerable expense, and some risk that may also heighten anxiety for the patient, their family, and their physicians (13).

To the best of our knowledge, no study to date has specifically investigated the outcome of people with normal LV volumes and ejection fraction with nonischemic patterns of LGE and no other manifestation of cardiac disease.

## Methods

### Patients

**Figure 1:**
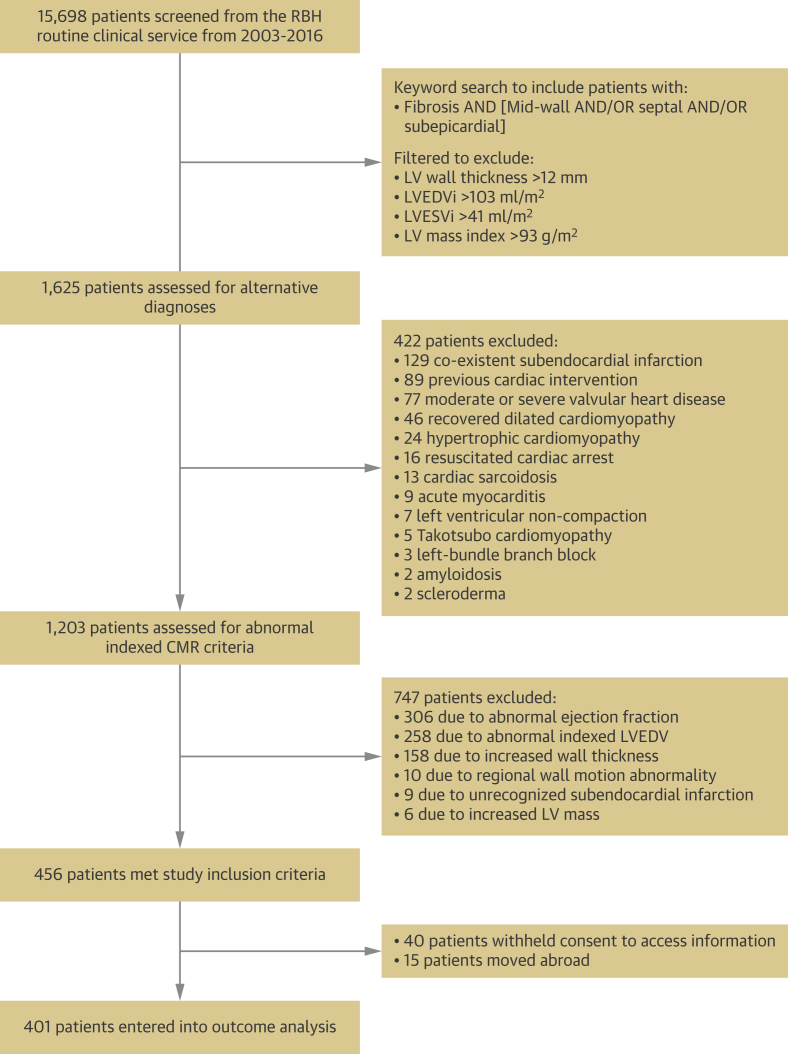
Patient Consort Flow Diagram Consort diagram shows the identification, inclusion and exclusion of the study population. CMR = cardiac magnetic resonance; RBH = Royal Berkshire Hospital; LV = left ventricular; LVEDVi = left ventricular end-diastolic volume index; LVESVi = left ventricular end-systolic volume index.

Patients referred for MRI between 2003 and 2016 with midwall and/or subepicardial LV myocardial fibrosis were identified. Exclusion criteria were applied in a stepwise approach (Figure 1). Filters were used to exclude all patients with LV wall thickness >12 mm, LV end-diastolic volume index (LVEDVi) >103 mL/m^2^, LV end-systolic volume index (LVESVi) >41 mL/m^2^ and LV mass index >93 g/m^2^ based on the upper 95% CI for men aged 20 years-29 years (14). All men aged >29 years and all women regardless of age would have indexed volumes below these upper thresholds. Then, patients with a potential reason for myocardial fibrosis, such as active myocarditis, sarcoidosis, previous chemotherapy, recovered dilated cardiomyopathy, or aortic stenosis were excluded. Similarly, we excluded patients with resuscitated cardiac arrest, where the need for implantable cardioverter-defibrillator (ICD) implantation for secondary prevention was already established. Patients with controlled hypertension receiving treatment with 1 or 2 antihypertensive agents at baseline were included, but not those with resistant hypertension (15). Patients with >50% stenosis in a major coronary artery, infarct pattern of LGE, left bundle branch block, coronary bypass grafting, or percutaneous intervention were also excluded. Finally, MRI data for remaining individuals were manually curated to exclude indexed LV values outside the appropriate age- and sex-adjusted normal ranges (14). In total, 456 patients met the stringent criteria to define a structurally normal heart (Figure 1).

In addition, a cohort of patients referred for MRI between 2003 and 2016 without myocardial fibrosis on LGE imaging (LGE-) provided a control cohort. These individuals were matched by scan indications. Inclusion and exclusion criteria were otherwise identical.

Patients provided written informed consent for the collection of clinical baseline and follow-up data as approved and directed by the National Research Ethics Service and received Institutional Board Approval by the Royal Brompton Hospital. The data supporting the findings of this study are available from the corresponding author upon request.

### MRI image acquisition and analysis

**Figure 2:**
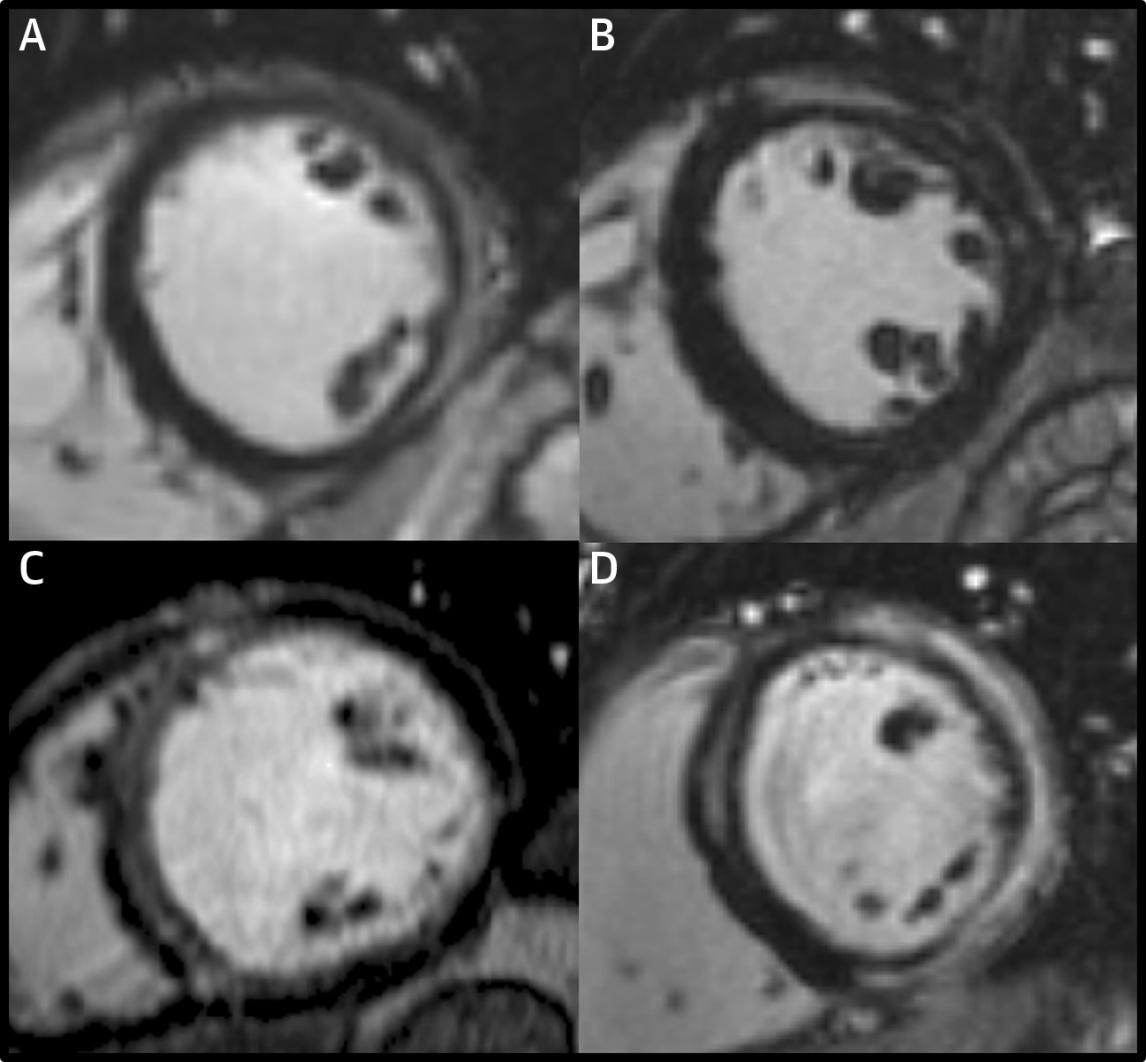
Typical MRI Late Gadolinium Enhancement Images Mid-ventricular short-axis views for 4 patients with normal indexed left ventricular volumes, wall thickness and ejection fraction demonstrating; **(A)** sub-epicardial enhancement in the inferolateral wall, **(B)** sub-epicardial enhancement in the anterolateral wall, **(C)** linear mid-wall enhancement in the septum, **(D)** mid-wall enhancement of the septum and sub-epicardial enhancement of the anterior, lateral and inferior walls. MRI = magnetic resonance.

MRI was performed on 1.5-T scanners (Sonata/Avanto) using a standardized protocol as previously described (Supplemental Methods). The presence, extent, and location (septal vs nonseptal) of midwall/subepicardial fibrosis was assessed by 2 independent expert readers who were blinded to clinical data, with a third senior expert providing adjudication if necessary (Figure 2). LGE mass (g) was quantified using the full-width at half-maximum technique and indexed as a percentage of LV mass (Supplemental Figure 1).

### Follow-up and outcomes

Patient follow-up was performed retrospectively by postal questionnaires, telephone interviews, and retrieval of information from primary care and hospital records. The presence of a family history of SCD was sought for all cases. Deaths were identified through the UK Health and Social Care Information Service. The prespecified primary outcome was a composite of actual or aborted SCD, as defined previously (16). The principal secondary outcome was all-cause mortality and a composite of CV mortality (SCD, heart failure, stroke, or thromboembolism) and unplanned CV hospitalization. Follow-up duration was calculated from the date of baseline MRI and censored at the first event or last patient contact. All clinical data were recorded in an electronic database and adjudicated by an independent committee of cardiologists blinded to MRI data. Cause of death was established from death certification and postmortems.

### Statistical analysis

Baseline characteristics (LGE+ vs LGE-) were compared using the Mann-Whitney *U* test or Fisher exact test. Patients with septal and nonseptal LGE were included in the septal group, given that septal and multiple patterns of LGE were previously recognized as the main drivers of arrhythmic risk (16). Medication was recorded at the time of baseline scan. Cumulative incidence curves were generated for outcomes with event times measured from the baseline MRI date. Associations between the location/extent of fibrosis and outcomes were analyzed using uni- and multivariable Cox proportional hazards modeling adjusted for known important predictors of outcome including age, sex, New York Heart Association (NYHA) functional class, and atrial fibrillation. The proportional hazards assumption was tested for the univariable and multivariable Cox models using plots of scaled Schoenfeld residuals and by testing the inclusion of an interaction term between time and each explanatory variable. To ensure that any observed effect was not driven by difference between LGE+/- cohorts at baseline, a sensitivity analysis was performed adjusting for additional potential confounders. Results are presented as HRs with 95% CIs. Statistical analyses were performed using Stata version 14 (StatCorp). A *P* value of < 0.05 was considered significant.

## Results

From 15,698 patients scanned with contrast in our institution from 2003 to 2016, 1,625 patients were identified for further evaluation into the LGE+ group (Figure 1). Of these, 422 (26%) were excluded due to coexistent pathology. Of the remaining 1,203 patients, 747 (62%) were excluded due to abnormalities of indexed LV measurements. As a result, 456 patients met inclusion and exclusion criteria. Of these, 40 withheld consent to follow-up and 15 had emigrated. Therefore, the LGE+ group consisted of 401 patients. A total of 347 LGE- patients were identified over the same period with matching scan indications, resulting in an overall cohort of 748 people.

### Baseline characteristics

The median age of the overall cohort was 50 y (interquartile range [IQR]: 38-61 years) and 287 (38%) were women (Table 1, Supplemental Figure 2). The main scan indications were chest pain (40%), palpitation/syncope (33%), or breathlessness (13%) (Supplemental Figure 3). A family history of SCD was reported for 35 patients (5%). Most patients were NYHA functional class I (83%) or II (15%). Overall, 25% were receiving beta-blockers, mostly for palpitation or chest pain, and 22% were receiving angiotensin-converting enzyme (ACE) inhibitors or angiotensin receptor blockers (ARBs), primarily for hypertension. The median LVEDVi was 77 mL/m^2^ (IQR: 66-85 mL/m^2^), LVESVi 25 mL/m^2^ (IQR: 21-31 mL/m^2^) and LVEF 66% (IQR: 62%-70%). LGE patterns were found to be septal in 69 (17%) patients, nonseptal in 305 (76%), and both septal and nonseptal in 27 (7%). The median LGE mass in the LGE+ group as a percentage of overall LV mass was 2.25% (IQR: 1.21%-4.14%).

**Table 1:** Baseline Patient Demographics for the 2 Study Groups Defined by the Presence or Absence of Nonischemic Late Gadolinium Enhancement (LGE+/-)

	All Patients (N = 748)	LGE		*P* Value
		No (n = 347)	Yes (n = 401)	
Age, y	50 (38-61)	49 (37-59)	51 (39-62)	0.11
Female	287 (38.4)	175 (50.4)	112 (27.9)	<0.0001
Body surface area, m^2^	1.9 (1.7-2.1)	1.9 (1.7-2.0)	1.9 (1.8-2.1)	<0.0001
Atrial fibrillation	42 (5.6)	17 (4.9)	25 (6.2)	0.43
Hypertension	173 (23.1)	53 (15.3)	120 (29.9)	<0.0001
Diabetes mellitus	56 (7.5)	22 (6.3)	34 (8.5)	0.27
Hypercholesterolemia	127 (17.0)	59 (17.0)	68 (17.0)	0.99
Current smoker	58 (7.8)	21 (6.1)	37 (9.2)	0.11
Cerebrovascular accident	15 (2.0)	2 (0.6)	13 (3.2)	0.010
Excess alcohol	98 (13.1)	50 (14.4)	48 (12.0)	0.32
Family history of sudden cardiac death	35 (4.7)	14 (4.0)	21 (5.2)	0.44
Medication				
ACE inhibitor	113 (15.1)	37 (10.7)	76 (19.0)	0.002
Beta blocker	186 (24.9)	66 (19.0)	120 (29.9)	<0.001
Angiotensin receptor blocker	55 (7.4)	13 (3.7)	42 (10.5)	<0.001
Anti-arrhythmia medication	36 (4.8)	15 (4.3)	21 (5.2)	0.56
New York Heart Association functional class				
I	624 (83.4)	320 (92.2)	304 (75.8)	<0.0001
II	120 (16.0)	27 (7.8)	93 (23.2)	
III	4 (0.5)	0 (0.0)	4 (1.0)	
Scan indication				
Chest pain	300 (40.1)	139 (40.1)	161 (40.1)	0.64
Palpitation or syncope	248 (33.2)	112 (32.3)	136 (33.9)	
Breathlessness	98 (13.1)	43 (12.4)	55 (13.7)	
Asymptomatic family screen	80 (10.7)	41 (11.8)	39 (9.7)	
Other	12 (1.6)	5 (1.4)	7 (1.7)	
Aortic assessment	10 (1.3)	7 (2.0)	3 (0.7)	
MRI parameters				
LVEDVi, mL/m^2^	77 (66-85)	74 (64-83)	79 (69-87)	<0.0001
LVESVi, mL/m^2^	25 (21-31)	24 (19-29)	26 (22-32)	<0.0001
LVEF, %	66 (62-70)	67 (63-72)	66 (62-69)	0.002
LV mass index, g/m^2^	63 (54-71)	59 (51-70)	66 (58-73)	<0.0001
RVEDVi, mL/m^2^	78 (68-89)	75 (64-87)	81 (71-91)	<0.001
RVESVi, mL/m^2^	30 (24-37)	29 (23-37)	31 (25-37)	0.03
RVEF, %	61 (56-66)	61 (56-66)	61 (56-65)	0.85
LGE, g	2.80 (1.50-5.25)	-	2.80 (1.50-5.25)	
LGE, %	2.24 (1.21-4.14)	-	2.24 (1.21-4.14)	
LGE >2.25%	202 (27.0)	-	202 (50.4)	

Values are median (interquartile range) or n (%).

ACE = angiotensin-converting enzyme; LGE = late gadolinium enhancement; LV = left ventricular; LVEDVi = left ventricular end-diastolic volume index; LVEF = left ventricular ejection fraction; LVESVi = left ventricular end-systolic volume index; MRI = magnetic resonance imaging; RVEDVi = right ventricular end-diastolic volume index; RVESVi = ventricular end-systolic volume index.

LGE+ patients were more likely to be men (*P <* 0.0001) with a history of controlled hypertension (*P <* 0.0001) and receiving treatment with a beta blocker or ARB (*P <* 0.001). NYHA functional class was also more likely to be II/III (*P <* 0.0001). LGE- patients were more likely to be women and have lower LVEDVi (*P <* 0.0001), and lower LV mass index (*P <* 0.0001) within the normal ranges. There were no significant differences between groups in age, comorbidity, or scan indication.

In the LGE+ group, those with nonseptal LGE were more likely to be men *(P =* 0.029) and to present with chest pain (*P <* 0.0001). Patients with septal LGE were more likely to be women *(P =* 0.029), to present with breathlessness or for familial cardiomyopathy screening (*P <* 0.0001), to have atrial fibrillation *(P =* 0.028), and to be prescribed an ACE inhibitor *(P =* 0.027). There were no significant differences between groups in age, baseline medical history, or medication.

### Primary outcome

**Figure 3:**
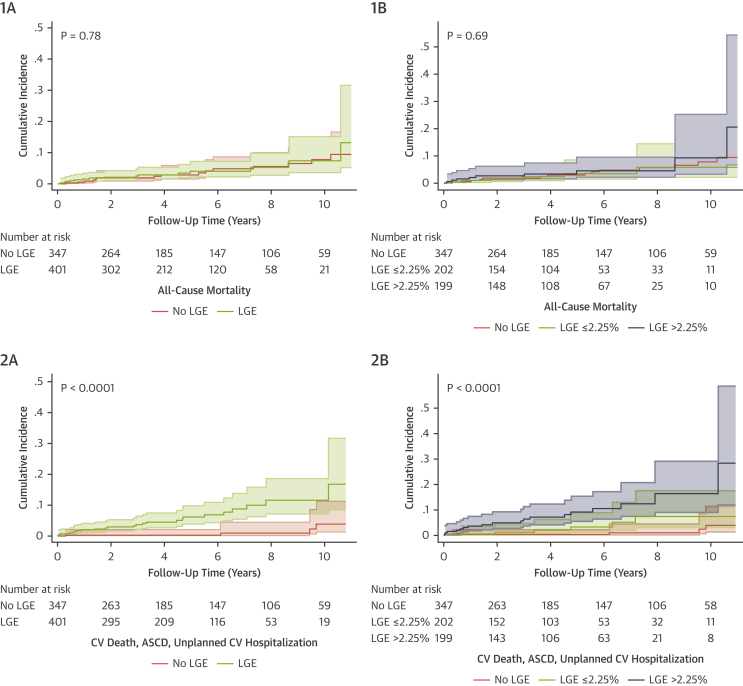
Cumulative Incidence Curves for Study Endpoints Cumulative incidence of **(1)** all-cause mortality and **(2)** CV death, aborted SCD and CV hospitalization stratified by **(A)** the presence or absence of LGE and **(B)** LGE extent above or below the median of 2.25% in this cohort with comparison to the LGE- group. CV = cardiovascular; SCD = sudden cardiac death; LGE = late gadolinium enhancement.

Over a median follow-up of 4.3 y (IQR: 2.1-6.5 years), only 1 patient (0.13% of the total cohort; 0.2% of the LGE+ group) met the primary outcome, presenting with an aborted SCD. This event occurred during the 11th year of follow-up in a patient who had a primary prevention ICD, basal-septal LGE, and no family history of SCD. The incidence rate per 100 patient-years in the LGE+ group was 0.05 (95% CI: 0.008-0.39) (Figure 3A).

In the LGE+ group, 11 patients had an ICD implanted for primary prevention without LV dilatation or reduction in LVEF; however, because of symptomatic episodes of nonsustained ventricular tachycardia (NSVT) (n = 7), asymptomatic NSVT with a family history of SCD (n = 1), or identification of clinically significant genetic variants with a family history of SCD (n = 3; lamin A/C, desmin, and desmoplakin) (Supplemental Table 1). Seven patients had an elective radiofrequency ablation procedure for NSVT. Thus, 18 LGE+ patients had an intervention for ventricular arrhythmia, of which 5 had septal LGE. In the LGE- group, 2 patients had a primary prevention ICD, and none had an intervention for ventricular arrhythmia.

### Secondary outcomes

#### All-cause mortality

There were 30 deaths during follow-up (4.0% of the total cohort), of which 2 were cardiovascular. The rate was similar for patients with (3.7%) and without (4.3%) LGE *(P =* 0.71). All-cause mortality was associated with patient age (HR: 2.04 per 10-year increase; 95% CI: 1.46-2.79; *P <* 0.001) and hypercholesterolemia (HR: 4.13; 95% CI: 2.01-8.47; *P <* 0.001) (Figure 3B, Table 2). The etiology for CV death was worsening heart failure from newly developed ischemic heart disease (both in men aged >70 years). Non-CV deaths included cancer (n = 18; 64%), pneumonia (n = 7; 25%), end-stage renal failure (n = 1), leukemia (n = 1), and motor neuron disease (n = 1). There was no association between LGE location or extent and all-cause mortality.

**Table 2:** Univariable Predictors of All-Cause Mortality (Presented as HRs and 95% CIs)

	HR (95% CI)	*P* Value
Age (per 10-y increase)	2.04 (1.49-2.79)	<0.0001
Female	1.35 (0.66-2.76)	0.42
Body surface area (m^2^)	0.38 (0.08-1.83)	0.23
Atrial fibrillation	1.46 (0.35-6.12)	0.61
Hypertension	0.79 (0.32-1.93)	0.61
Diabetes mellitus	0.38 (0.05-2.82)	0.35
Hypercholesterolemia	4.13 (2.01-8.47)	<0.001
Current smoker	0.38 (0.05-2.82)	0.35
Cerebrovascular accident	0.00 (0.00-0.00)	1.00
Excess alcohol	1.77 (0.76-4.14)	0.18
Family history of sudden cardiac death	0.62 (0.08-4.55)	0.64
Medication		
ACE inhibitor	1.35 (0.55-3.29)	0.52
Beta blocker	0.76 (0.31-1.87)	0.56
ARB	0.40 (0.05-2.90)	0.36
Anti-arrhythmia medication	0.62 (0.08-4.56)	0.64
New York Heart Association functional class		
I	Ref.	
II/III	2.33 (1.07-5.10)	0.03
MRI parameters		
LVEDVi (mL/m^2^)	0.98 (0.95-1.00)	0.08
LVESVi (mL/m^2^)	0.96 (0.91-1.01)	0.13
LVEF (%)	1.02 (0.95-1.08)	0.63
LV mass index (g/m^2^)	0.99 (0.96-1.02)	0.48
RVEDVi (mL/m^2^)	0.98 (0.96-1.00)	0.11
RVESVi (mL/m^2^)	0.97 (0.93-1.01)	0.09
RVEF (%)	1.04 (0.99-1.09)	0.16
LGE presence	1.11 (0.53-2.30)	0.78
LGE extent:		
None	Ref.	0.55
≤2.25%	0.88 (0.34-2.30)	
>2.25%	1.33 (0.58-3.09)	

ARB = angiotensin receptor blocker; other abbreviations as in Table 1.

#### CV death, aborted SCD, and unplanned hospitalization

During follow-up, there were 2 CV deaths and 73 CV hospital admissions (9.7% of the total cohort), of which 25 (34%) were unplanned. Twenty-one of 401 LGE+ patients (5.2%) experienced this composite outcome compared with 4 of 347 LGE- patients (HR: 7.22; 95% CI: 4.26-21.17; *P <* 0.0001). Indications for unplanned admissions included a first-confirmed episode of myocarditis (n = 7), palpitation due to NSVT (n = 5), cerebrovascular accident (CVA) (n = 4), pacemaker implantation (n = 3), atrial fibrillation (n = 2), myocardial infarction (n = 2), and pulmonary embolism (n = 2). Of those admitted for investigation of palpitation with NSVT, all were LGE+ and underwent radiofrequency ablation and/or ICD implantation as outlined above. In patients with LGE and an event, 79% had an LGE mass index above the median of 2.25% (LGE+ HR: 11.27; 95% CI: 3.73-34.07 vs LGE- HR: 3.55; 95% CI: 0.99-12.75; *P <* 0.0001) (Figure 3D). Other variables that showed an association on univariable analysis (Table 3) included prior CVA (HR: 8.26; 95% CI: 2.86-23.85; *P <* 0.0001) and prescription of beta-blockers (HR; 3.09; 95% CI: 1.47-6.49; *P =* 0.003) or other antiarrhythmic medication (HR: 5.70; 95% CI: 2.31-14.06; *P <* 0.001).

**Table 3:** Univariable and Multivariable Analyses for the Composite Secondary Outcome of CV Death, Aborted SCD and Unplanned CV Hospitalization

	Univariable		Multivariable	
	HR (95% CI)	*P* Value	HR (95% CI)	*P* Value
Age, y	1.07 (0.84-1.37)	0.58	1.04 (0.81-1.33)	0.77
Female	0.62 (0.27-1.42)	0.26	1.00 (0.42-2.37)	1.00
Body surface area, m^2^	3.48 (0.77-15.62)	0.10		
Atrial fibrillation	3.48 (1.20-10.06)	0.02	3.62 (1.20-10.98)	0.02
Hypertension	1.52 (0.69-3.36)	0.30		
Diabetes mellitus	1.30 (0.39-4.31)	0.67		
Hypercholesterolemia	1.01 (0.38-2.66)	0.98		
Current smoker	3.77 (1.60-8.88)	0.002		
Cerebrovascular accident	8.26 (2.86-23.85)	<0.0001		
Excess alcohol	1.27 (0.48-3.34)	0.63		
Family history of sudden cardiac death	0.66 (0.09-4.84)	0.68		
Medication				
ACE inhibitor	1.78 (0.76-4.19)	0.19		
Beta blocker	3.09 (1.47-6.49)	0.003		
ARB	0.87 (0.21-3.66)	0.85		
Anti-arrhythmia medication	5.70 (2.31-14.06)	<0.001		
New York Heart Association functional class				
I	Ref.		Ref.	
II/III	2.26 (0.99-5.14)	0.05	1.47 (0.61-3.52)	0.39
MRI parameters				
LVEDVi, mL/m^2^	1.00 (0.97-1.03)	0.87		
LVESVi, mL/m^2^	1.01 (0.96-1.06)	0.59		
LVEF, %	0.96 (0.90-1.02)	0.19		
LV mass index, g/m^2^	0.99 (0.96-1.02)	0.36		
RVEDVi, mL/m^2^	1.00 (0.98-1.03)	0.82		
RVESVi, mL/m^2^	1.00 (0.96-1.04)	0.95		
RVEF, %	1.01 (0.96-1.06)	0.74		
LGE presence	7.22 (2.46-21.17)	<0.001	7.16 (2.30-22.28)	0.001
LGE extent:				
None	Ref.			
≤2.25%	3.55 (0.99-12.75)			
>2.25%	11.27 (3.73-34.07)	<0.0001		

CV = cardiovascular; SCD = sudden cardiac death; other abbreviations as in Table 1.

In a multivariable analysis adjusting for age, sex, atrial fibrillation, and NYHA functional class, LGE presence remained associated with this secondary composite outcome (HR: 7.16; 95% CI: 2.30-22.58; *P =* 0.001). There was no strong evidence that the proportional hazards assumption was violated for any explanatory variable in the models. Additionally, we performed a sensitivity analysis (including hypertension, current smoker, previous CVA, and any medication use) to confirm that LGE presence remained associated with this endpoint after adjustment for imbalances of between groups (HR: 6.51; 95% CI: 2.06-20.58; *P =* 0.001) (Supplemental Table 2).

### Genetic sequencing

Thirteen of 39 LGE+ patients (33%) with a family history of cardiomyopathy as the scan indication (although phenotypically unaffected) were subsequently found to have rare variants in genes associated with cardiomyopathy (Supplemental Table 1). Of these patients, 3 had ICDs implanted for primary prevention; however, none met the primary outcome, and only 2 met the composite secondary outcome. Sensitivity analysis confirmed that the primary study findings were not altered significantly by the inclusion or exclusion of this group.

## Discussion

Management of patients with midwall/subepicardial fibrosis in the setting of normal LV volumes and ejection fraction is a clinical conundrum due to the lack of data on how to manage and advise such patients. This is the first study to investigate the prognostic significance of nonischemic patterns of LGE in patients with normal LV volumes and ejection fraction. Overall, there was a low burden of major arrhythmic events during a median follow-up of 4.3 years. All-cause mortality was driven primarily by age-related disease and was not associated with presence or absence of LGE (Central Illustration). However, there was an increase in the burden of unplanned CV hospitalization among patients with LGE, particularly among those with a greater volume of LGE, independent of age.

**Central Illustration:**
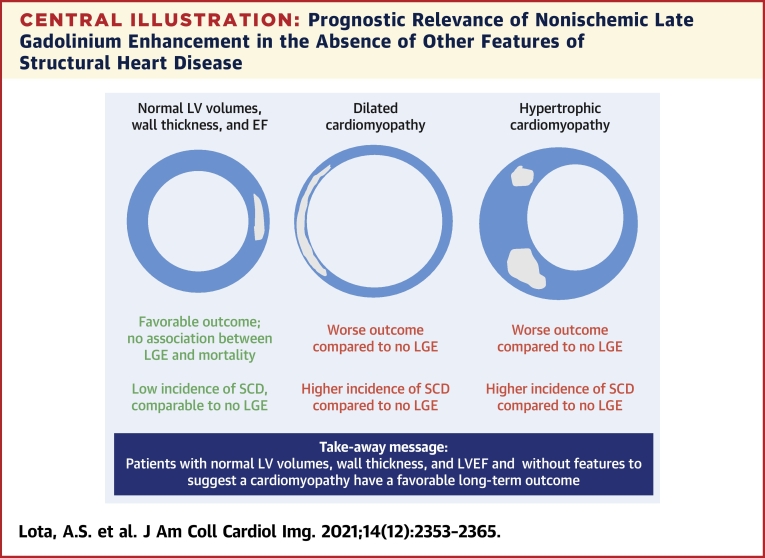
Prognostic Relevance of Nonischemic Late Gadolinium Enhancement in the Absence of Other Features of Structural Heart Disease Nonischemic late gadolinium enhancement by cardiovascular magnetic resonance in the absence of other risk factors, such as LV dilatation, increased wall thickness, reduced LVEF or a family history of cardiomyopathy, is associated with a favorable long-term outcome.

SCD remains a major public health issue with devastating impact. Traditional approaches to risk stratification are imprecise and reliant on LVEF. However, the vast majority of SCDs occur among patients either not diagnosed with heart disease (45% of patients) or with a history of heart disease but LVEF > 40% (40% of patients) (17). As emphasized in the 2017 American Heart Association guidelines for management of patients with ventricular arrhythmias, there is unmet need to improve the identification of individuals without ventricular dysfunction who are at risk for SCD. In our cohort, there was a low overall burden of major arrhythmic events. The only aborted SCD event occurred in a patient with septal LGE. The underlying arrhythmia that triggered an appropriate shock was monomorphic ventricular tachycardia initiated by a premature ventricular ectopic couplet occurring during sinus tachycardia. This suggests that patients with incidental and otherwise unexplained, nonischemic patterns of LGE do not require ICD implantation if LV volumes, wall thickness, and ejection fraction are all within normal limits. Furthermore, this observation supports the notion that the genesis of ventricular arrhythmia is dependent on the presence of multiple factors, of which structural substrate is just one component. Our data suggest that replacement myocardial fibrosis in a nonischemic pattern in the absence of other risk factors, such as LV dilatation, reduced LVEF, or a family history of cardiomyopathy, is not a marker of high risk even over an extended period of follow-up.

Our results should be considered in the context of other studies investigating the significance of LGE in patients with no known CV disease. In 939 patients (median age: 76 years) from the Age, Gene/Environment Susceptibility (AGES)–Reykjavik study, incidental infarct-pattern LGE was detected in 17% and this was independently associated with all-cause mortality (8). Similarly, in 310 patients with LVEF > 50%, infarct-pattern LGE predicted cardiac transplantation and all-cause mortality (10). In a study of 44 patients with myocardial infarction, the presence of even small amounts of LGE (<2% mean LV mass) was associated with a 7-fold increase in the HR for major adverse cardiac events, and remained an independent predictor when adjusted for LVEF (11). In our study, mortality was associated with increasing age and hypercholesterolemia rather than presence/absence of LGE; the low CV mortality in our cohort may reflect the stringent exclusion of patients with coronary artery disease. We confirmed the excellent negative predictive value of an entirely normal MRI scan reported in a previous study of 225 patients with clinically suspected myocarditis, in which no patient with a normal MRI had a major cardiovascular event over >4 years of follow-up (18).

In a subgroup analysis of the AGES–Reykjavik cohort, 54 patients (6%) were identified with “major” nonischemic patterns of LGE attributed to myocarditis, infiltrative cardiomyopathy, or pathological hypertrophy (19). LGE was associated with a primary composite endpoint of all-cause mortality and heart failure hospitalization (HR: 3.2), and it was associated with a poorer outcome than infarct-pattern LGE, which was present in 211 (23%) patients (HR: 2.3). This study emphasized the prognostic significance of etiologically heterogeneous, nonischemic scar in a relatively small number of individuals with normal LVEF (median: 62%) but later in life with greater levels of comorbidity compared to our cohort.

Understanding of the dose-response relationship between LGE extent and SCD in any cardiac disease remains challenging. In our study, patients with higher volumes of LGE were more likely to have CV hospitalization, mainly due to concerns about myocarditis or palpitation/arrhythmia. However, there were insufficient numbers of events to evaluate LGE extent as a continuous variable. Nevertheless, abnormal test results are likely to increase surveillance and it is unclear to what extent the test result rather than underlying disease drove hospitalization rates, including ICD implantation and ablation procedures. The relationship between greater medication use and the composite secondary outcome may reflect a higher burden of disease; however, prescribing bias should also be considered. Ambulatory electrocardiograph monitoring was not performed routinely; therefore, the true burden of subclinical arrhythmia is unknown.

In our cohort, underlying etiology of LGE in patients with otherwise normal LV volumes and ejection fraction was often uncertain, reflecting real-world clinical practice. Lateral free-wall LGE is often ascribed to a previous, potentially silent, episode of myocarditis (20). The prevalence of myocarditis is likely to be globally underestimated. Myocarditis accounts for 11.6% of all SCD found in patients who are <35 y of age, and yet is only detected on 2% of SCD postmortem studies, suggesting widespread under-recognition of this potentially arrhythmogenic substrate (21,22). Explanations for lateral wall predilection include greater susceptibility of watershed territories to parvovirus B19-mediated endothelial dysfunction and polyserositis from the adjacent pericardium. In our cohort, most patients had lateral-wall LGE, and of these, a greater percentage were men and presented with chest pain, all typical of myocarditis.

Lateral-wall LGE may be seen with other pathologies such as lamin cardiomyopathy, early presentations of left-dominant forms of arrhythmogenic cardiomyopathy, Duchenne’s muscular dystrophy cardiomyopathy, or Anderson-Fabry disease before LV hypertrophy. Although it is possible that many of our patients had a remote episode of myocarditis, other genetic forms of cardiomyopathy are also a consideration (12). In our cohort, the main revised diagnosis downstream was of gene carrier status. No other patients developed a new diagnosis on follow-up that may have accounted for their initial presentation.

Whereas genetic cardiomyopathies are generally associated with adverse outcomes, the event rate in the preclinical phase of disease with normal LV volumes and ejection fraction is low, as observed in our cohort. Lamin cardiomyopathy is strongly associated with malignant arrhythmia and a prognostic model of 4 risk factors was recently proposed consisting of LVEF <45%, NSVT, male sex, and lamin A/C mutation and presence of a pathogenic lamin A/C variant. However, no malignant ventricular arrhythmia occurred in patients with <2 risk factors (23). There is also emerging evidence of an overlap between myocarditis and arrhythmogenic ventricular cardiomyopathy. However, lamin cardiomyopathy and arrhythmogenic ventricular cardiomyopathy are both progressive diseases, associated with poor outcome in the setting of even mild LV dysfunction; therefore, they require close follow-up.

### Study limitations

The patient cohort was enrolled from a single center; therefore, it may be susceptible to referral bias. However, our referral base for MRI is broad and includes a range of secondary and tertiary centers. After several levels of filtering, the final LGE+ study cohort comprised 401 patients from a pool of 15,698 patients. This reflects the stringency of our algorithm. Ethnicity is known to influence LV measurements, particularly wall thickness, and the findings of this study are mostly applicable to Caucasians.

The normal range criteria used in this study were described in a study of 120 healthy people aged 20- to 80-years old (14). Other groups have sought to characterize normal ranges in larger cohorts, such as the UK Biobank (24). In this study, after multiple exclusion steps, 802 (16.2%) healthy participants were identified with upper LVEDVi cutoffs of 110 mL/m^2^ (men) and 94 mL/m^2^ (women) irrespective of age. LV papillary muscles were included in the LV cavity; therefore, whereas ventricular volumes tended to be larger as expected, this did not materially affect interpretation of our data. The full-width at half-maximum method was used to quantify LGE, which may underestimate LGE quantity but provides the highest intra- and interobserver reproducibility (25,26).

Follow-up MRI data were not available to evaluate the presence of adverse remodeling in LGE+ patients. MRI assessment of extracellular volume, a preclinical biomarker of reactive interstitial fibrosis, using T1 mapping was not available at the beginning of the study. Retrospective MRI assessment of myocardial strain may help to confirm whether diastolic dysfunction was a significant contributor to NYHA functional class III status identified in 3.5% of LGE+ patients, although left atrial size and when available plasma brain natriuretic peptide were normal, suggesting that noncardiac comorbidities may have contributed to symptoms. Similarly, routine genetic sequencing might have provided additional insight into etiology.

Given the identification of a single aborted SCD event, it is challenging to confirm whether this represents a true negative study outcome or a type II error. Future work is required with larger patient groups, but this initial study suggests this patient group is at a very low risk of SCD.

## Conclusions

Our data provide new information on the prognostic significance of nonischemic patterns of LGE in a large, well-characterized cohort of patients with normal LV volumes and ejection fraction. We demonstrate, for the first time, that there is a reassuringly low risk of actual or aborted SCD in this population. All-cause mortality was driven primarily by age-related disease and was not associated with the presence of LGE. These findings do not support aggressive medical management or the routine use of ICD implantation within this cohort.

Perspectives **COMPETENCY IN MEDICAL KNOWLEDGE:** The identification of nonischemic (mid-wall/subepicardial) LGE in the absence of other risk factors, such as LV dilatation, reduced LVEF or a family history of cardiomyopathy, is not a marker of increased SCD risk or all-cause mortality. Therefore, aggressive medical therapy or routine use of ICD implantation are not advised in this cohort.

**TRANSLATIONAL OUTLOOK:** Further studies are needed to verify the generalizability of these observations to other populations and to develop more personalized SCD risk assessment strategies for patients identified with nonischemic patterns of myocardial fibrosis.

## Funding Support and Author Disclosures

Supported by the Cardiovascular Research Centre at Royal Brompton and Harefield NHS Foundation Trust, the National Heart and Lung Institute, Imperial College London, the Alexander Jansons Myocarditis UK and the Wellcome Trust. Dr Lota was funded by a British Heart Foundation Clinical Research Training Fellowship (FS/17/21/32712). Dr Prasad has received funding from the Alexander Jansons Myocarditis UK, Rosetree Trust, British Heart Foundation, the Medical Research Council and the Coronary Artery Disease Research Association; and has received honoraria for talks from Bayer Schering. Dr Pennell has received research support from Siemens, has performed consultancy work for Bayer and Apotex and is a stockholder of CVIS. Dr Cleland has received non-financial research support from BSCI and Medtronic and speaker honoraria from Medtronic. Dr Cook is a consultant for Illumina and a shareholder in Enleofen Bio. All other authors have reported that they have no relationships relevant to the contents of this paper to disclose.
