# Supplementary material for: Prognostic Significance of Nonischemic Myocardial Fibrosis in Patients With Normal LV Volumes and Ejection-Fraction
Source: JACC Cardiovasc Imaging. 2021 Dec;14(12):2353–65. doi: 10.1016/j.jcmg.2021.05.016 (PMC8648892; doi:10.1016/j.jcmg.2021.05.016)
Supplement: Supplemental Data [file mmc1.doc]

**Online appendix**

[CMR Image Acquisition and Analysis: 2](#__RefHeading___Toc62051350)

[Follow-up and Outcomes 2](#__RefHeading___Toc62051351)

[Additional References 3](#__RefHeading___Toc62051352)

[Supplementary Figure 1. Method for late gadolinium enhancement quantification 4](#__RefHeading___Toc62051353)

[Supplementary Figure 2: Histogram of cohort age distribution 5](#__RefHeading___Toc62051354)

[Supplementary Figure 3. CMR scan indications 6](#__RefHeading___Toc62051355)

[Supplementary Table 1. List of individual genetic variants detected 7](#__RefHeading___Toc62051356)

[Supplementary Table 2. Sensitivity analysis 9](#__RefHeading___Toc62051357)

**Methods**

CMR Image Acquisition and Analysis:

CMR was performed on 1.5Tesla scanners (Sonata/Avanto, Siemens, Germany) using a standardized protocol with 0.1mmol/kg of gadolinium contrast agent, either Magnevist or Gadovist (Bayer, Germany).(1,2) Inversion-recovery gradient echo sequences were used with images repeated in two separate phase-encoding directions in multiple orthogonal views to exclude artefacts. LV volumes, LV ejection fraction and mass were measured using dedicated software (CMRtools) and indexed to body surface area.(3) Blood pool thresholding was used to delineate and exclude the papillary muscles from ventricular volumes. LGE was considered as present when seen in two orthogonal planes, in both phase-encoding directions and extending beyond the focal ventricular insertion points but excluding right ventricular septo-marginal trabeculae. The full-width at half maximum (FWHM) method was used to quantify LGE (CMR42, Circle Cardiovascular Imaging Inc, Calgary, Canada).(4) This method estimates the mass of myocardium with signal intensity >50% of the maximally enhanced myocardium defined by the user. Using the same method, we have previously shown the absolute mean difference between operators in LGE quantification to be 0.87% (intraclass correlation coefficient: 0.87).(5)

Follow-up and Outcomes

SCD was defined as unexpected death <1 hour of the onset of cardiac symptoms in the absence of progressive cardiac deterioration, during sleep, or ≤24 hours of last being seen alive.(6) Aborted SCD was defined as an appropriate ICD shock for ventricular arrhythmia, a non-fatal episode of ventricular fibrillation, or sustained VT with haemodynamic compromise requiring cardioversion.(7)

Additional References

1. Halliday BP, Gulati A, Ali A et al. Association Between Midwall Late Gadolinium Enhancement and Sudden Cardiac Death in Patients With Dilated Cardiomyopathy and Mild and Moderate Left Ventricular Systolic Dysfunction. Circulation 2017;135:2106-2115.

2. Gulati A, Jabbour A, Ismail TF et al. Association of fibrosis with mortality and sudden cardiac death in patients with nonischemic dilated cardiomyopathy. Jama 2013;309:896-908.

3. Maceira AM, Prasad SK, Khan M, Pennell DJ. Normalized left ventricular systolic and diastolic function by steady state free precession cardiovascular magnetic resonance. J Cardiovasc Magn Reson 2006;8:417-26.

4. Amado LC, Gerber BL, Gupta SN et al. Accurate and objective infarct sizing by contrast-enhanced magnetic resonance imaging in a canine myocardial infarction model. Journal of the American College of Cardiology 2004;44.

5. Halliday BP, Baksi AJ, Gulati A et al. Outcome in Dilated Cardiomyopathy Related to the Extent, Location, and Pattern of Late Gadolinium Enhancement. JACC Cardiovasc Imaging 2019;12:1645-1655.

6. Hicks KA, Tcheng JE, Bozkurt B et al. 2014 ACC/AHA Key Data Elements and Definitions for Cardiovascular Endpoint Events in Clinical Trials: A Report of the American College of Cardiology/American Heart Association Task Force on Clinical Data Standards (Writing Committee to Develop Cardiovascular Endpoints Data Standards). Circulation 2015;132:302-61.

7. Buxton AE, Calkins H, Callans DJ et al. ACC/AHA/HRS 2006 key data elements and definitions for electrophysiological studies and procedures: a report of the American College of Cardiology/American Heart Association Task Force on Clinical Data Standards (ACC/AHA/HRS Writing Committee to Develop Data Standards on Electrophysiology). Journal of the American College of Cardiology 2006;48:2360-96.

Supplementary Figure 1. Method for late gadolinium enhancement quantification

A mid-ventricular, short-axis late gadolinium enhancement image is shown for reference (left) with quantification performed using the full-width at half maximum method (right). The green line defines the epicardial border, the red line defines the endocardial border, the pink area outlines a reference region of LGE and the yellow region outlines the area of LGE quantified.

**
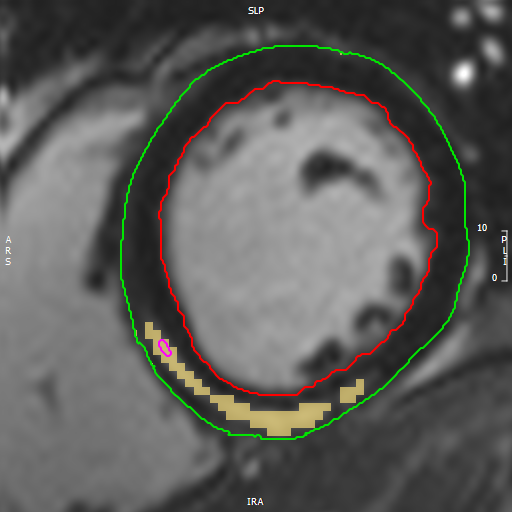

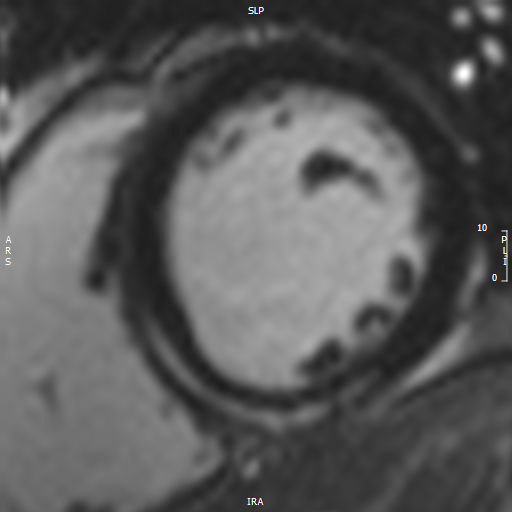
**

Supplementary Figure 2: Histogram of cohort age distribution

Histogram of patient age by the presence/absence of LGE at time of baseline CMR (p=0.11).


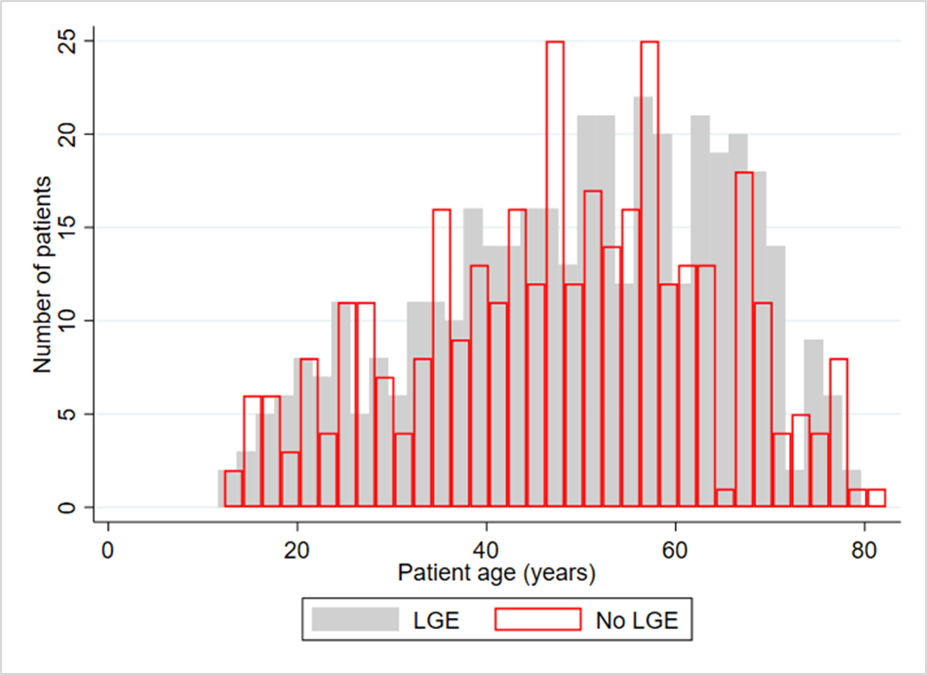


Supplementary Figure 3. CMR scan indications

Pie chart showing CMR scan indications across the whole cohort (n=748)


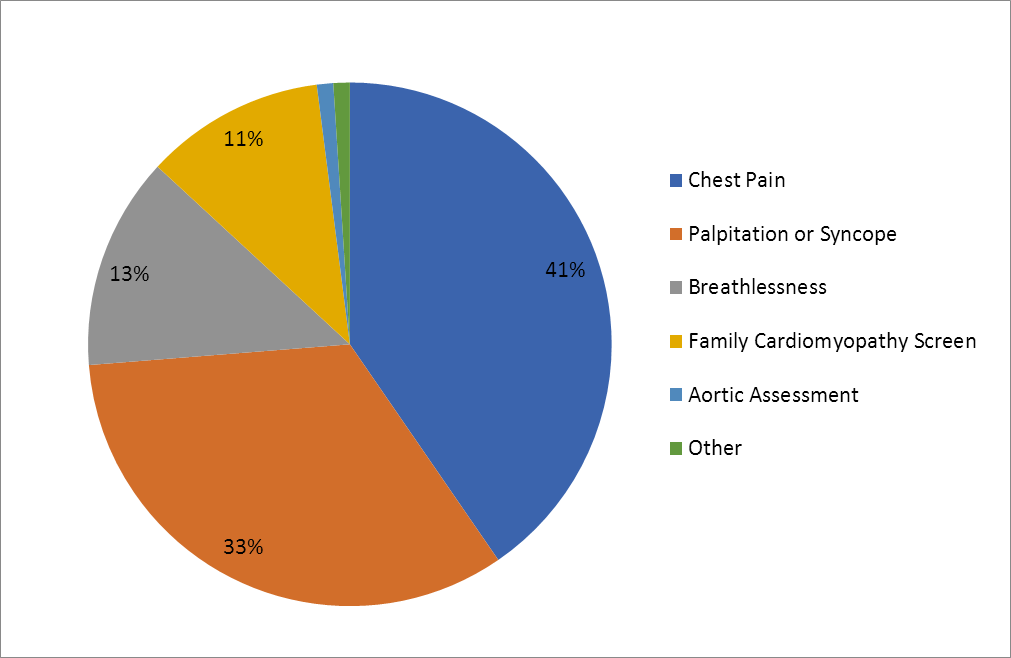


Supplementary Table 1. List of individual genetic variants detected through routine clinical evaluation

*ACMG class was determined using CardioClassifier15 followed by manual curation of segregation, de novo and functional data from the literature and ClinVar. P = pathogenic, LP = likely pathogenic, VUS = variant of uncertain significance, PM = pathogenic moderate. For three further patients, the medical records referred to clinical genetic testing that had reportedly identified rare variants in cardiomyopathy-associated genes, but it was not possible to identify precise details of the variant in the medical record. These were variants in DSG2, DSP & LMNA (1 variant per patient).

Supplementary Table 2. Sensitivity analysis

Sensitivity analysis of the composite secondary outcome (CV death, ASCD and unplanned CV hospitalisation) adjusting for potential confounders (hypertension, current smoker, prior CVA and any medication use).

| **Variable** | **HR (95% CI)** | **P** |
| --- | --- | --- |
| LGE presence | 6.51 (2.06, 20.58) | 0.001 |
| Age (years) | 1.04 (0.78, 1.39) | 0.77 |
| Female | 1.07 (0.45, 2.55) | 0.87 |
| Atrial fibrillation | 3.16 (1.05, 9.51) | 0.04 |
| Hypertension | 0.90 (0.36, 2.26) | 0.83 |
| Current smoker | 3.20 (1.32, 7.75) | 0.01 |
| Cerebrovascular accident | 4.82 (1.57, 14.85) | 0.006 |
| Any medication use (ACE/BB/ARB/anti-arrhythmic) | 1.53 (0.65, 3.64) | 0.34 |
| **New York Heart Association** |  |  |
| Class I | Reference group |  |
| Class II/III | 1.16 (0.47, 2.89) | 0.74 |
